# Mechanisms of Desiccation Tolerance: Themes and Variations in Brine Shrimp, Roundworms, and Tardigrades

**DOI:** 10.3389/fphys.2020.592016

**Published:** 2020-10-23

**Authors:** Jonathan D. Hibshman, James S. Clegg, Bob Goldstein

**Affiliations:** ^1^Department of Biology, The University of North Carolina at Chapel Hill, Chapel Hill, NC, United States; ^2^Bodega Marine Laboratory, University of California, Davis, Davis, CA, United States; ^3^Lineberger Comprehensive Cancer Center, The University of North Carolina at Chapel Hill, Chapel Hill, NC, United States

**Keywords:** desiccation tolerance, anhydrobiosis, *Artemia*, tardigrade, nematode, *C. elegans*, trehalose, LEA proteins

## Abstract

Water is critical for the survival of most cells and organisms. Remarkably, a small number of multicellular animals are able to survive nearly complete drying. The phenomenon of anhydrobiosis, or life without water, has been of interest to researchers for over 300 years. In this review we discuss advances in our understanding of protectants and mechanisms of desiccation tolerance that have emerged from research in three anhydrobiotic invertebrates: brine shrimp (*Artemia*), roundworms (nematodes), and tardigrades (water bears). Discovery of molecular protectants that allow each of these three animals to survive drying diversifies our understanding of desiccation tolerance, and convergent themes suggest mechanisms that may offer a general model for engineering desiccation tolerance in other contexts.

## Introduction

Water is essential for life. The metabolism of life occurs in an aqueous environment, and the biological constituents of cells – DNA, RNA, protein, and membranes – are all unstable during desiccation. However, some animals defy this reasoning, surviving in the near-absence of water by entering a quiescent state and later resuming activity upon rehydration. Antonie van Leeuwenhoek first described reviving dehydrated “animalcules” from the sediment in his gutter in 1702 (Keilin, 1959). Other investigators followed in his footsteps to conduct similar experiments, yet the seemingly miraculous survival and revitalization of desiccated animals remained controversial (Keilin, 1959). The writing of 18^*th*^ century biologist Lazzaro Spallanzani captures the astonishing nature of this feat of survival:

*To affirm that a being, whose animation has been suspended for an immoderate length of time, even for years, can, in virtue of certain conditions, be revived, has so singular and paradoxical an appearance, that reason finds it repugnant to admit the fact. But to produce an animal which has been stiff and motionless, withered, disfigured, and contracted, utterly incapable of any corporeal function… and, by a particular treatment, to make it renovate every action that it could perform before; …not only will it bear perfect conviction to the mind that it has come from a state which, if it was not death, certainly cannot be called life, but that it again lives as completely as before its animation was suspended or destroyed. Some animals in the world enjoy this wonderful prerogative (Translated from original Italian in* Spallanzani and Dalyell, 1803).

The suspended state between life and death that Spallanzani describes is now known as anhydrobiosis, or “life without water.” Some organisms, like the African killifish whose embryos can survive evaporation of ephemeral pools, prevent water loss by insulating cells with cuticle or other barriers that serve to trap water inside (Podrabsky et al., 2001). These are considered to be desiccation resistant organisms. However, certain multicellular animals are able to survive even in the absence of almost all intracellular water. These are true anhydrobiotes. So far, anhydrobiotic survival has been observed in tardigrades, roundworms, rotifers, and select arthropods including brine shrimp and chironomid midges. Upon dehydration, these animals significantly reduce metabolic activity and mount a regulated response to produce cellular protectants. Researchers have sought to identify protective mechanisms in many of the known anhydrobiotic organisms, and each organism has revealed new aspects of the biology of desiccation tolerance. Much can be learned by comparing work across these organisms and identifying common themes, and variations on those themes, that allow for the remarkable ability of animals to survive complete desiccation.

**FIGURE 1:**
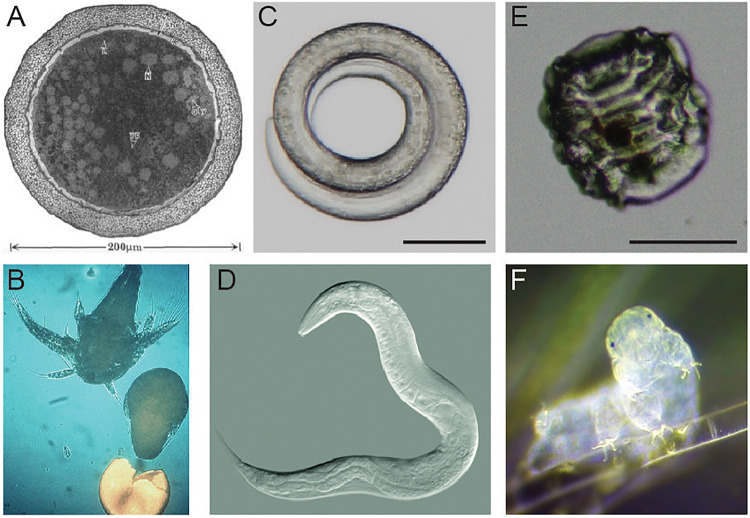
Examples of *Artemia*, *Caenorhabditis elegans*, and a tardigrade. **(A)** A transmission electron micrograph of an encysted embryo of *Artemia franciscana*. This image is reproduced with permission from Clegg et al., 1999. **(B)** A nauplius larva of *Artemia* hatches. Photo credit: Patrick Sorgeloos. **(C)** A desiccated dauer larva of *C. elegans* in the typical curled form. Scale bar = 50 μm. Photo credit: J. Hibshman. **(D)** A reproductive *C. elegans* adult. Photo credit: B. Goldstein. **(E)** A tardigrade (*Hypsibius exemplaris*) in the anhydrobiotic tun state. Scale bar = 50 μm. Photo credit: J. Hibshman. **(F)** An active tardigrade (*Hypsibius exemplaris*). Photo credit: Sinclair Stammers.

This review summarizes major advances in our knowledge of the desiccation responses of three anhydrobiotic animals: brine shrimp (genus *Artemia*), roundworms (with a focus on *Caenorhabditis elegans*), and tardigrades. Figure 1 shows examples of each animal in anhydrobiotic and active states. During desiccation, *Artemia*, roundworms, and tardigrades all undergo substantial physical and physiological changes. *Artemia* are able to survive dehydration as diapausing embryos (Figure 1A). Dauer larvae of the nematode *C. elegans* responds to desiccation by curling into a circular shape (Figure 1C). Desiccated tardigrades enter the contracted and shriveled tun state (Figure 1E). We focus on *Artemia*, *C. elegans*, and tardigrades because there is some overlap in features of desiccation tolerance between these organisms, and there are also meaningful differences in survival strategies. Importantly, each of these organisms is a true anhydrobiote, surviving in the absence or near absence of intracellular water. Each has been reported to have virtually no water when dried. *Artemia* have as little as 2% water relative to the dry weight of cysts, and desiccated *C. elegans* and tardigrades each retain only 2–3% of the water that was present in their active states (Crowe, 1972; Clegg, 1974; Clegg and Cavagnaro, 1976; Erkut et al., 2011).

One key distinction between these animals is differences in the timeline of responsiveness to desiccation. Tardigrades and roundworms (nematodes) that are exposed to desiccation conditions are able to survive as larvae or adults, whereas *Artemia* are only able to survive desiccation as embryonic cysts. The decision to make a desiccation resistant cyst is made in the previous generation rather than by the animal that will itself experience drying. A second difference between these three animals is the correlation between diapause and desiccation tolerance. Diapause is a state of suspended development in response to environmental cues. *Artemia* cysts enter an embryonic diapause, and *C. elegans* survive desiccation in dauer diapause (Drinkwater and Crowe, 1987; Erkut et al., 2011). Tardigrades, on the other hand, enter the quiescent tun state and survive desiccation without an alternative developmental trajectory. Although some species of tardigrades can form diapausing cysts, encystment does not ensure desiccation tolerance (Bertolani et al., 2004). Thus, a comparative organismal approach to desiccation tolerance should nuance our views and allow for identification of both unique and conserved mechanisms of anhydrobiosis.

The approaches researchers have employed in studying *Artemia*, nematodes, and tardigrades have been somewhat complementary and revealed different aspects of the physiology and molecular details of desiccation resistance. In this review we explore the molecules and mechanisms of desiccation tolerance, highlight common themes, and suggest open questions whose answers will be instrumental to the advancement of our understanding of desiccation tolerance.

## Artemia

Some of the earliest work on desiccation tolerance was carried out on encysted embryos of brine shrimp (genus *Artemia*). Johannes Albertus Schlosser is credited with the discovery and initial description of *Artemia* (Sliggers and Engelmann, 2015). Schlosser originally found the “brine worms” in salt water along the coast in Lymington, England in 1755 (see Sliggers and Engelmann, 2015). *Artemia* are important in aquaculture as food for fish, and they have been marketed as “sea monkeys” as pets for children (Sorgeloos, 1980; Berenbaum, 1999). The commercial uses of *Artemia* and the related commercial interest in developing culture methods fortuitously facilitated the use of these animals as a laboratory model for the study of diapause and resistance to stresses including anoxia, various forms of radiation, and desiccation (MacRae, 2016). Multiple species of *Artemia* are able to survive these stresses as diapausing cysts (Hengherr et al., 2011b).

*Artemia* present an interesting case study of environmental regulation of diapause and stress resistance; they have two alternative developmental trajectories. If environmental conditions are favorable, females retain their developing embryos and release free-swimming nauplius larvae (ovoviviparous development); however, when environmental conditions are poor, females will instead release gastrula-stage embryos (oviparous development) that diapause until conditions amenable to hatching and development are present (see Figures 1A,B) (Sorgeloos, 1973; Drinkwater and Crowe, 1987; Liang and MacRae, 1999; Nambu et al., 2004, 2008). The late gastrula stage embryos arrest development concurrent with a drop in intracellular pH, and are surrounded by a thick, multi-layered, protective shell (Busa et al., 1982; Busa and Crowe, 1983; Liu et al., 2009). These dormant cysts are metabolically inactive and can withstand a variety of insults including anoxia, osmotic stress, temperature extremes, high levels of radiation, and desiccation (Clegg, 1967, 1997, 2005; MacRae, 2016). To date, studies of *Artemia* desiccation resistance have converged on a few key features that contribute to their desiccation tolerance.

### Cyst Shell

The shell of a dormant cyst is the interface between the organism and the environment. Some animals protect against dehydration by retaining water. *Artemia* cysts are anhydrobiotic, meaning they lose nearly all intracellular water. Thus, the shell of *Artemia* cysts does not prevent water loss. If the shell does not function to maintain water, how does it contribute to desiccation resistance? The shell slows the rate of water loss from embryos, and by limiting the rate of dehydration, the shell may provide more time for the embryo to mount responses to desiccation such as a gradual slowdown of metabolism and production of protectants required to ultimately survive desiccation (Clegg, 2005).

The shell is synthesized from secreted products of the shell glands of the female, and consists of three layers: an outer cuticle, a middle alveolar layer, and the innermost fibrous layer (Morris and Afzelius, 1967; Anderson et al., 1970; Liu et al., 2009). These layers are porous to water and CO_2_, but are able to serve as a barrier against many toxins (MacRae, 2016). Perhaps most importantly in the context of desiccation, the shell offers mechanical stability and protection from external damage to the embryos. Depletion of a shell gland-specifically expressed gene (SGEG) compromises the structure of the shell. Upon desiccation or osmotic stress, cysts with these disrupted shells do not maintain a spherical shape, but instead collapse (Liu et al., 2009). Similarly, disruption of the shell by knockdown of SGEG1 or SGEG2 increases sensitivity to UV radiation, freezing, and heat stress (Dai et al., 2011). Other studies have found similar results, with manual decapsulation (removal of the outermost layer of the shell) sensitizing cysts to UV irradiation, heat, and desiccation (Sorgeloos et al., 1977; Tanguay et al., 2004; Clegg, 2005; Liu et al., 2009). The protective ability of cysts’ shells therefore extends beyond desiccation and serves as a reminder of the complex environment in which *Artemia* live and the variety of concurrent stresses they may encounter and survive.

### Trehalose Synthesis

Trehalose is a non-reducing disaccharide of glucose that has been demonstrated to be protective in diverse anhydrobiotes (Crowe, 2002). Although trehalose is not synthesized in vertebrates, it can improve desiccation tolerance when provided to human cells (Guo et al., 2000; Collins and Clegg, 2004; Argüelles, 2014). *Artemia* embryos developing into cysts synthesize trehalose in large quantities of up to around 15% of the embryo’s dry weight, while non-dormant embryos have virtually no measurable trehalose (Clegg, 1965). Trehalose has been implicated as a way to replace water as cells dehydrate, a model known as the water replacement hypothesis (Crowe and Clegg, 1973; Clegg et al., 1982; Crowe, 2002).

### Metabolic Adaptations

Metabolic activity is negligible in dormant cysts; however, in preparation for encystment there are a couple of metabolic changes that occur. Aside from the significant upregulation of trehalose, there is increased production of glycerol and glycogen (Clegg, 1962, 1965). It is possible that glycerol contributes to hatching of cysts, and glycerol has proven effects in freeze-tolerance (Clegg, 1964). Glycogen also provides a means of osmotic regulation by storing individual molecules of glucose in long branched chains. It remains unclear the extent to which production of glycerol and glycogen might be mechanisms for osmo-regulation and desiccation survival versus an energy store to be utilized during recovery. Trehalose may also be converted to these molecules – possibly contributing to osmo-protective and energetic functions (Clegg, 1964).

### Artemin

Artemin is a ferritin homolog in *Artemia* and has demonstrated activity as a molecular chaperone (Chen et al., 2007). Artemin is significantly upregulated in dormant cysts and has been reported to comprise 7–13% of the cyst’s total soluble protein (De Graaf et al., 1990; King et al., 2014). By sequence homology, artemin is similar to ferritin, which assembles into a 24-mer structure that functions as a cage that holds iron in its center, acting to regulate iron homeostasis. Artemin similarly oligomerizes as a 24-mer, but does not bind iron. Each monomer contains a tail that protrudes into the center of the barrel such that iron is excluded (Chen et al., 2003, 2007). Despite losing the iron-binding function of ferritin, artemin has been demonstrated to have chaperone activity: it can prevent aggregation of citrate synthase and other proteins *in vitro* (Chen et al., 2007; Hu et al., 2011; Shahangian et al., 2011). Thus, the massive quantity of artemin produced in dormant cysts likely functions to prevent or reverse desiccation-induced protein aggregation and proteotoxicity via this chaperone function.

### Small Heat Shock Proteins

The protein p26 is a small heat shock protein, with a characteristic alpha-crystallin domain flanked by disordered N-terminal and C-terminal regions (Liang et al., 1997a,b; Laksanalamai and Robb, 2004). p26 is transcriptionally upregulated during cyst formation, and expression levels decline rapidly upon rehydration and larval development (Liang and MacRae, 1999). Normally, environmental cues like low temperature exposure or changes in salinity are required for embryos to exit diapause and resume development (Drinkwater and Crowe, 1987). Knockdown of p26 by RNAi before diapause entry results in spontaneous exit from diapause, and reduces the hatching rate of cysts exposed to freezing and desiccation, as well as heat shock (King and MacRae, 2012). p26 can also confer resistance to oxidative stress and desiccation when expressed in heterologous systems (Collins and Clegg, 2004; Ma et al., 2005). Other small heat shock proteins were subsequently discovered in *Artemia*. Hsp21 and Hsp22 are both upregulated in cysts and have demonstrable chaperone activity (Qiu and MacRae, 2008a,b). Cysts express p26 at much higher levels than Hsp21 and Hsp22, and Hsp21 and Hsp22 are seemingly less important for survival of stress than is p26 (King et al., 2013).

Although small heat shock proteins are typically only 15–42 kDa, they often oligomerize to form larger complexes (Kim et al., 1998; Haslbeck, 2002). Likewise, p26, although only 20.8 kDa as a monomer, forms higher order complexes. It was reported that *in vivo* p26 oligomerizes into complexes of around 27 monomers – over 700 kDa (Liang et al., 1997a). Analysis of p26 heterologously expressed in bacteria or cultured mammalian cells confirms oligomerization, albeit to a lesser extent, with the most common oligomers containing 5 monomers (Sun et al., 2004). Assembly into higher order oligomers was shown to depend on the N-terminal tail of the protein (Sun et al., 2004). Other targeted mutations within the alpha-crystallin domain can also disrupt oligomerization, suggesting a role for the crystallin domain in the oligomerization interface (Sun et al., 2006).

Small heat shock proteins can localize in different parts of cells. p26 has been detected in both the cytoplasm and the nucleus. Antibody staining revealed formation of distinct p26 foci within the nucleus (Liang et al., 1997a; Willsie and Clegg, 2001). The nucleus may be the predominant site of action as the protein is trafficked from the cytoplasm to the nucleus during diapause and in response to anoxia, pH, or temperature changes (Clegg et al., 1995; Jackson and Clegg, 1996; Willsie and Clegg, 2001). When p26 is translocated into the nucleus during diapause and stress, it associates with the nuclear matrix, although other localization sites are possible (Willsie and Clegg, 2002).

The activity of small heat shock proteins as chaperones is likely to be non-enzymatic, as they associate with misfolded proteins to prevent further denaturation and aggregation during desiccation stress (Jakob et al., 1993; Chakrabortee et al., 2012). sHSPs have been dubbed holdases by nature of their activity to surround misfolded proteins and prevent aggregation until an ATP-driven heat shock protein like Hsp70 can bind and refold the protein; indeed, p26 and Hsp70 were shown to co-localize in the nucleus (Willsie and Clegg, 2002).

### Hsp70

The 70 kDa heat shock proteins (Hsp70s) are a family of ATP-dependent chaperones essential for protein folding. Knockdown of one family member, Hsp70, in *Artemia* sensitizes developing nauplii to heat stress and infection with the bacterium *Vibrio campbellii* (Iryani et al., 2017; Junprung et al., 2019). Knockdown of Hsp70 has a modest effect on survival of desiccated and frozen cysts (Iryani et al., 2020). J-domain proteins are a prominent family of co-chaperones that assist Hsp70 with protein folding and can confer client specificity (Kampinga and Craig, 2010). Two J-domain proteins have been identified in *Artemia*: ArHsp40 and ArHsp40-2 (Jiang et al., 2016; Rowarth and MacRae, 2018b). Knockdown of either of the genes encoding these proteins reduces the percentage of cysts that are able to hatch following freezing and desiccation (Rowarth and MacRae, 2018a). Developmentally, ArHsp40 and ArHsp40-2 reach their peak expression levels in embryos exiting diapause (Jiang et al., 2016; Rowarth and MacRae, 2018b). This timeline is consistent with p26 preventing protein misfolding and aggregation during desiccation, and with Hsp70 acting in concert with ArHsp40 and ArHsp40-2 during early stages of recovery to refold proteins.

### LEA Proteins

Synthesis of intrinsically disordered proteins (IDPs) is a common mechanism contributing to anhydrobiosis in a variety of organisms. One of the most prominent examples of an IDP is the family of late embryogenesis abudant (LEA) proteins. LEA proteins were originally identified in plants, but subsequently have been found in multiple animal species, including *Artemia* (Dure et al., 1981; Baker et al., 1988; Browne et al., 2002; Hand et al., 2006; Warner et al., 2010, 2012; Hatanaka et al., 2013). There are seven classes of LEA proteins that have been defined in plants, but most animals have only group 3 LEA proteins (Battaglia et al., 2008). *Artemia* contain LEA proteins representing groups 1, 3, and 6, making them apparently unique to date among animals (Wu et al., 2011; Janis et al., 2017). These LEA proteins and the mRNAs encoding them are enriched in diapausing cysts (Hand et al., 2006; Sharon et al., 2009; Warner et al., 2010, 2012; Boswell et al., 2014b).

Significant upregulation of LEAs in diapause-destined *Artemia* embryos suggests a possible function in desiccation resistance. A common experimental strategy to test for LEA function has been to assess chaperone activity or protective capacity in heterologous or *in vitro* contexts. LEA proteins from *Artemia* are capable of protecting liposomes, stabilizing membranes, attenuating protein aggregation, and conferring resistance to osmotic and desiccation stresses (Sharon et al., 2009; Li et al., 2012; Marunde et al., 2013; Hand and Menze, 2015; Moore and Hand, 2016; Moore et al., 2016; Czernik et al., 2020). While such studies highlight potential functions of LEA proteins, the endogenous roles of these proteins in *Artemia* have been less well-studied. One study using RNAi knockdown revealed that a group 1 LEA protein is also *necessary* for desiccation tolerance in *Artemia* cysts (Toxopeus et al., 2014).

One of the potentially major challenges facing animals undergoing desiccation is the number and diversity of subcellular components that must be protected. The study of *Artemia* LEA proteins provides insight into the importance of subcellular compartmentalization of the desiccation response. AfrLea3m was the first LEA from an animal that was shown to localize to mitochondria, when bioinformatic predictions of subcellular localization were confirmed with heterologous expression and studies in human cell culture (Menze et al., 2009). Ensuing subcellular fractionation experiments identified different LEA proteins in cytosolic, mitochondrial, and nuclear fractions (Warner et al., 2012). Visualizing endogenous LEA proteins with immunofluorescence confirmed the mitochondrial localization of AfrLEA3m and revealed that AfrLEA2 has a primarily cytoplasmic localization, with some faint nuclear localization (Boswell and Hand, 2014). The distribution of different LEA proteins to different parts of a cell may explain the diversity of LEAs that are expressed and could represent a strategy for targeted protection of subcellular compartments. For example, the mitochondrial localization of AfrLEA3m suggests a role in preservation of mitochondria during stress, protecting the integrity of the organelle to provide energy for mounting a desiccation response and to fuel recovery of the cell when rehydrated.

### Regulation of Desiccation Resistance

Desiccation tolerance mechanisms must be coordinated in order to ensure that each cell of a cyst has the required suite of protectants. Many stress responses are under a coherent program of transcriptional regulation (Vihervaara et al., 2018). Identification of “master regulators” of the protectants required for desiccation tolerance suggests that the desiccation response is also subject to coordinated control and could inform the search for additional, novel protectants also controlled by these regulators. In *Artemia*, the heat shock factor 1 (Hsf1) regulates expression of Artemin and the small heat shock proteins p26, Hsp21, and Hsp22 (Tan and MacRae, 2018, 2019). RNAi knockdown of Hsf1 reduces viability of cysts and sensitizes them to stress, including desiccation (Tan and MacRae, 2018). Hsf1 is one transcription factor that can serve as a regulator of the desiccation response, but there are likely other regulators (transcriptional and post-transcriptional) that are involved. It is further possible that transcriptional co-activators like *p8* may provide specificity to the transcriptional initiation of diapause and desiccation resistance (Qiu and MacRae, 2007; Qiu et al., 2007; Lin et al., 2016).

### Lessons From *Artemia*

The unique life cycle of *Artemia* has allowed for its use as a model in the study of desiccation tolerance and other forms of stress tolerance. The ability to reproducibly generate diapausing cysts in the lab has offered a window to study mechanisms of both diapause and resistance to extremes. While this system has been valuable for answering both of these questions, some ambiguity remains between adaptations of cysts that are necessary for initiating or maintaining diapause versus those that are involved in desiccation tolerance *per se*. The overlapping nature of diapause and desiccation resistance likely reflects the ecological contexts in which such stress responses have evolved. Tolerance of high saline environments has likely facilitated co-adaptation to desiccation stress. Although it is unclear what the initial selective pressure was that drove the evolution of encystment – desiccation resistance or high salt – the unique adaptations of *Artemia* likely reflect the complexity of the natural environment.

The earliest studies of *Artemia* focused on metabolic adaptations and proteins that were upregulated during initiation of diapause. The identification of trehalose as a prominent metabolite produced in cysts would become an important theme amongst desiccation tolerant organisms. In fact, it has been commonplace to ask early on in the study of any new anhydrobiote the extent to which its tolerance depends on trehalose. Further study of trehalose has also led to theories and mechanisms of desiccation tolerance including the water replacement hypothesis and the cellular vitrification model, both of which will be discussed in more detail below (Clegg et al., 1982; Green and Angell, 1989). The early focus on metabolic studies has given way to a more protein-centric approach amongst *Artemia* researchers. This focus has promoted discoveries like assembly of p26 into oligomers and the structural nature of artemin (Sun et al., 2004; Hu et al., 2011). Evidence for endogenous function of protectants is scarce (see Table 1), as the more common approach has been to analyze protective capacity *in vitro* or in heterologous systems.

**TABLE 1:** Comparison of molecular strategies for desiccation survival between *Artemia*, *C. elegans*, and tardigrades. Genes or proteins implicated in desiccation tolerance are shown.

	*Artemia*	*C. elegans*	Tardigrades
*Mechanical stabilization*	Cyst shell	Cuticle	Cuticle

*Metabolic changes*	Production of glycerol and glycogen	Glyoxylate shunt (***icl-1***)	

*Trehalose*	Yes	Yes (***tps-1*, *tps-2***)	Variable

*LEA proteins*	Groups **1**,3,6	Group 3 (***lea-1***,***dur-1***)	Group 3

*Heat shock proteins*	**Hsp70**, J-domain proteins (**ArHsp40**, **ArHsp40-2**)	***hsp-70***	Hsp70
*Small heat shock proteins (sHSPs)*	**p26**, Hsp21, Hsp22	***F08H9.3***,***F08H9.4***, *hsp-12.6*	Hsp27, Hsp30c

*Oxidative stress response*		Superoxide dismutase (***sod-1***)	Increased superoxide dismutase activity
		Glutathione peroxidase (***gpx-2***,***gpx-6***,***gpx-7***)	Increased peroxidase activity
		Catalase (***ctl-1***)	

*Fatty acid desaturation*		**Δ**6 desaturase (***fat-3)*** **Δ**5 desaturase (***fat-4***) Δ9 desaturase (***fat-5***, ***fat-6***,***fat-7***)	

*Polyamine synthesis*		Ornithine decarboxylase (***odc-1***) Spermidine synthase (***spds-1***)	

*Xenobiotic detoxification*		Cadmium responsive (***cdr-2***, ***cdr-3***) Glyoxylase III (***djr-1.1***, ***djr-1.2***)	

*Others*	Artemin (Ferritin) Heat shock factor 1 **(Hsf1)**	Calexcitins (***cex-1***, ***cex-2***) Trypsin like protease (***try-5***) UDP-Glucuronosyltransferase (***ugt-1***) Serine threonine kinase (***C04G2.2***) TRP channel (***osm-9***) Notch ligand (***osm-11***) Patched related protein (***daf-6***)	Tardigrade-specific disordered proteins (**CAHS**, **SAHS**, MAHS)

*In bold are those that have been demonstrated to have an effect on desiccation tolerance in the endogenous, organismal context. References are included in the textual descriptions for each item delineated in the table. Genes included from *C. elegans* without specific textual references are described in Erkut et al. (2013).*

Many questions remain about the protection of cells of the *Artemia* embryo. For example, encysted embryos are able to develop into pre-nauplii without cell or nuclear divisions (Clegg, 1966; Olson and Clegg, 1978). Therefore, it would appear that the internal components of the nuclei, most notably the DNA, would have to be protected. It is also possible that a period of development without DNA replication allows time for DNA repair (McLennan, 2009). This unique biology of *Artemia* embryos remains a topic for future research. The discoveries made from studying mechanisms of desiccation resistance in *Artemia* have synergized with studies in nematodes, the animals to which we now turn our attention.

## Nematodes

Nematodes were first reported to survive desiccation in the 1700s (Needham, 1743). John Needham described adding water to a blighted grain of wheat and seeing fiber-like worms come to life:

*…to my great surprise, these imaginary fibers, as it were, instantly separated from each other, took life, moved irregularly, not with a progressive, but twilling motion, and continued so to do for the space of nine or ten hours…* (Needham, 1743)

Since Needham’s discovery of the reanimation of “fibers,” later to be identified as larvae of the plant pathogenic nematode *Anguina tritici*, desiccation tolerance has been described in numerous species of nematodes, including *Aphelenchus avenae*, *Plectus murrayi*, *Panagrolaimus superbus*, *Ditylenchus dipsaci*, and *C. elegans* (Keilin, 1959; Madin and Crowe, 1975; Wharton, 1996; Perry, 1999; Shannon et al., 2005; Adhikari et al., 2009; Erkut et al., 2011; Tyson et al., 2012). For some of these worms, like *A. avenae* and *C. elegans*, intracellular water content has been specifically measured, showing depletion to anhydrobiotic levels with loss of nearly all intracellular water (Crowe et al., 1977; Erkut et al., 2011). Although many nematodes have been studied in the context of desiccation resistance, we focus here on *C. elegans* and supplement that analysis with other species in some instances.

*Caenorhabditis elegans* is a common model organism with well-developed methods and resources for genetics, cell biology, and imaging that can be used to understand the physiology of stress responses (Brenner, 1974). Of the anhydrobiotic metazoans, *C. elegans* offers the most resourced molecular toolkit (Wharton, 2011; Dickinson et al., 2013; Erkut and Kurzchalia, 2015; Nance and Frøkjær-Jensen, 2019). Although other nematodes like *A. avenae* have been long appreciated as having anhydrobiotic abilities, it was only relatively recently that *C. elegans* was shown to be a true anhydrobiote, surviving desiccation after losing more than 98% of their body water content (Madin and Crowe, 1975; Erkut et al., 2011). Some nematodes can withstand desiccation at multiple life stages, but *C. elegans* is only resistant to desiccation when in the dauer state, a specialized stress-resistant alternative larval stage in which the worms stop feeding (Cassada and Russell, 1975; Erkut et al., 2011). Similar to the diapause of *Artemia* cysts, *C. elegans* enter dauer diapause in response to deteriorating environmental conditions by undergoing metabolic and morphological changes including synthesis of a specialized cuticle, radial constriction, and formation of a plug over the mouth (Riddle et al., 1981; Hu, 2007).

*Caenorhabditis elegans* has been a productive model for identifying genes involved in anhydrobiosis. Studies have mostly taken two approaches: (1) expression profiling to identify genes whose mRNA abundances change during desiccation and (2) disruption of candidate genes’ functions to identify genes that are required for desiccation tolerance. These approaches have identified molecules that suggest a striking diversity of physiological adaptations to desiccation, some prominent examples of which we discuss in the sections below.

### Trehalose Synthesis

Trehalose accumulates in several anhydrobiotic nematode species (Madin and Crowe, 1975; Loomis et al., 1980; Behm, 1997; Shannon et al., 2005). In *C. elegans*, the upregulation of trehalose synthesis during gradual drying is essential for worms to survive desiccation (Erkut et al., 2011). *C. elegans* trehalose synthesis is a two-step process that generates trehalose from glucose and UDP-glucose, involving trehalose 6-phosphate synthase genes (*tps-1* and *tps-2*) and the subsequent dephosphorylation of trehalose 6-phosphate by a protein encoded by *gob-1* (Kormish and McGhee, 2005). Deletion of both *tps* genes limits the ability of *C. elegans* to synthesize trehalose and retain appropriate membrane lipid packaging, and to survive desiccation (Erkut et al., 2011; Abusharkh et al., 2014). Trehalose also functions in other contexts including aging and starvation resistance, suggesting that its protective role may extend beyond desiccation tolerance (Honda et al., 2010; Hibshman et al., 2017; Seo et al., 2018).

### Metabolic Adaptations

**FIGURE 2:**
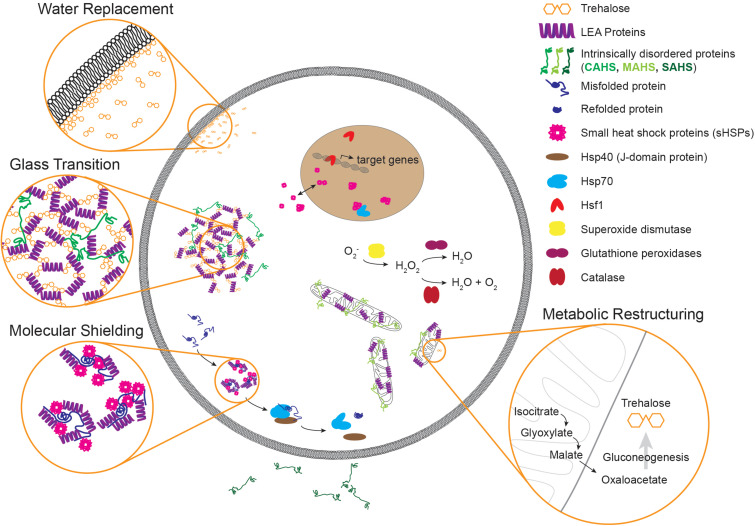
An illustration of molecular components and mechanisms implicated in desiccation tolerance. Common molecules that contribute to desiccation tolerance are depicted in subcellular compartments where they have been shown or are suggested to function. Some components like small heat shock proteins can occupy multiple subcellular compartments, as evidenced by p26 from *Artemia*. Desiccation can lead to protein misfolding and aggregation. During times of desiccation, proteins like sHSPs and LEAs may limit aggregation, and during recovery Hsp70 may refold these proteins to restore proteostasis. During gradual drying, metabolic preparations occur like upregulation of the glyoxylate shunt and production of trehalose. Three common models of protection during desiccation are depicted as well. Although highlighted in three separate insets, these mechanisms likely have a large degree of overlap. Note that components are not to scale. LEA, late embryogenesis abundant; CAHS, cytosolic abundant heat soluble; MAHS, mitochondrial abundant heat soluble; SAHS, secretory abundant heat soluble; Hsp40, 40 kilodalton heat shock protein; Hsp70, 70 kilodalton heat shock protein; Hsf1, heat shock factor 1.

During anhydrobiosis, metabolism grinds to an apparent halt (Crowe et al., 1977). However, during gradual desiccation there are a number of preparatory metabolic adaptations that occur. Entry into the dauer state drives restructuring of metabolic flux (O’Riordan and Burnell, 1989, 1990; Burnell et al., 2005). One of the most significant metabolic changes with a demonstrated effect on desiccation tolerance is an upregulation of the glyoxylate shunt (Madin et al., 1985; Erkut et al., 2016). The glyoxylate shunt, involving isocitrate lyase and malate synthase enzymes, is an alternative to the TCA cycle that converts isocitrate to succinate and malate (Braeckman et al., 2009). In *C. elegans*, isocitrate lyase and malate synthase enzyme activities are performed by a single protein, ICL-1, that carries out both of these functions (Liu et al., 1995). Depletion of *icl-1* by RNAi sensitizes *C. elegans* to desiccation (Erkut et al., 2016). The malate produced from glyoxylate shunt activity provides an energetic input for gluconeogenesis and ultimately the synthesis of trehalose during preconditioning for desiccation (Figure 2) (Erkut et al., 2016). Thus, it is likely that metabolic adaptations such as upregulation of the glyoxylate shunt primarily function to fuel production of downstream protectants, rather than having an intrinsic protective ability. In short, the glyoxylate shunt promotes the conversion of fatty acids into carbohydrates like trehalose that can function as protectants.

### LEA Proteins

The first report of an animal LEA protein was in the nematode *Aphelenchus avenae*, where it was shown to be upregulated in response to desiccation (Browne et al., 2002, 2004). *C. elegans* contains two group 3 LEA protein-encoding genes, *lea-1*, and *dur-1* (dauer up-regulated) (Browne et al., 2004; Gal et al., 2004; Erkut et al., 2013). Each of these genes’ expression is induced by desiccation, and knockdown reduces the ability of worms to survive desiccation (Gal et al., 2004; Erkut et al., 2013). The initial discovery of an LEA protein in *A. avenae* was followed by a flurry of papers reporting LEA proteins in a wide variety of animals (including *Artemia*, as already discussed) (Gal et al., 2004, 2006; Tunnacliffe et al., 2005; Hand et al., 2006; Kikawada et al., 2006). Despite identification of many animal LEAs, most studies of these proteins in the organismal context are limited to expression profiling and rare cases of functional validation by knockdown. Like *Artemia* LEA proteins, nematode LEA proteins have also been studied through heterologous expression or *in vitro* studies. An LEA protein from *A. avenae* can prevent protein aggregation in multiple contexts, and can modestly improve osmotolerance of human cells (Goyal et al., 2005; Chakrabortee et al., 2007; Liu et al., 2011). LEA-1 from *C. elegans* can also reduce aggregation of proteins and can improve bacterial resistance to oxidative stress and desiccation (Liu et al., 2019).

Late embryogenesis abudant proteins are highly hydrophilic and intrinsically disordered in solution, confounding traditional intuition that proteins must fold into functional forms (Tunnacliffe and Wise, 2007). However, the structure of LEA proteins is highly responsive to conditions within the cell. Using the LEA1 protein from the nematode *Aphelenchus avenae* as a model, Goyal et al. (2003) demonstrated an increase in secondary structure during desiccation. Specifically, LEA proteins fold into amphipathic alpha helices in the absence of water. This has also been shown for *C. elegans* LEA-1 (Liu et al., 2019). Folding into alpha helices during desiccation (or similar experimentally simulated conditions) has proven to be a common attribute of a variety of LEA proteins and model peptides from animals including nematodes and *Artemia* (Tolleter et al., 2007; Li and He, 2009; Boswell et al., 2014a; Hand and Menze, 2015; LeBlanc et al., 2019). Amphipathic alpha helices are thought to stabilize molecular interactions by maintaining appropriate charge contact sites via opposite charged and non-polar faces of the helix (Giménez-Andrés et al., 2018). LEA proteins may also act as an interface between air and water to prevent protein aggregation (Yuen et al., 2019). The amphipathic structure of LEA proteins allows for diverse functions. LEA proteins act as “molecular shields” by limiting aggregation of misfolded proteins, contribute to membrane stabilization, and are constituents of biological glasses as dehydrating cells vitrify (Hand et al., 2011).

### Heat Shock Proteins

In a microarray study measuring transcriptional changes during desiccation, the small heat shock protein-encoding gene *F08H9.4* was the most upregulated gene, increasing mRNA level over 700-fold (Erkut et al., 2013). Two other sHSP-encoding genes, *F08H9.3* and *hsp-12.6*, were also upregulated in response to desiccation. When tested for a function in desiccation survival, an *hsp-12.6* mutant did not affect survival, but mutations in either *F08H9.3* or *F08H9.4* reduced desiccation tolerance, with *F08H9.3* having a more pronounced effect (Erkut et al., 2013). In *C. elegans hsp-70* is transcriptionally upregulated during desiccation and required for wild-type levels of desiccation tolerance (Erkut et al., 2013). A role for *hsp-70* in worm desiccation survival highlights again the role of proteostasis in desiccation tolerance.

### Oxidative Stress Response

A common feature of desiccation is oxidative stress (França et al., 2007). Reactive oxygen species (ROS) can accumulate during desiccation, damaging proteins, membranes, and DNA. Multiple nematode species upregulate antioxidant response genes during desiccation (Adhikari et al., 2009; Tyson et al., 2012). For example, in *C. elegans* the expression levels of several antioxidant genes increase during gradual desiccation, and there is functional evidence that a superoxide dismutase gene (*sod-1*), several glutathione peroxidases (*gpx-2*, *gpx-6*, *gpx-7*) and catalase (*ctl-1*) are required for desiccation survival, suggesting that upregulation of these genes (all except *ctl-1* have increased expression during desiccation) is an adaptive physiological response to gradual desiccation (Erkut et al., 2013).

### Fatty Acid Desaturation

Multiple fatty acid desaturases are transcriptionally upregulated in response to desiccation (Erkut et al., 2013). Additionally, multiple genes involved in fatty acid desaturation are necessary for full desiccation tolerance. These include Δ9 fatty acid desaturases (*fat-5*, *fat-6*, and *fat-7*), Δ6 desaturase (*fat-3*), and Δ5 desaturase (*fat-4*). Of note, the ω3 desaturase, *fat-1*, does not impact desiccation survival, suggesting that arachidonic acid may play a particular role in desiccation resistance (Erkut et al., 2013). How fatty acid desaturation leads to desiccation resistance is an open question. It is possible that breakdown of fatty acids could provide an energy reserve during a time of limited activity of the TCA cycle and oxidative phosphorylation. It is also possible that desaturated fatty acids in lipid bilayers could contribute to membrane fluidity (Crowe et al., 1989). A third possibility is that various lipid species may undergo peroxidation, functioning as sinks for ROS during desiccation.

### Regulation of Desiccation Resistance

*Caenorhabditis elegans* relies on gradual preconditioning to upregulate the protectants required for desiccation survival (Erkut et al., 2011). Thus, *C. elegans* (and other multicellular animals) must have mechanisms to recognize gradual loss of water and to induce the responses described above. Even animals like fruit flies that cannot survive total desiccation can sense and respond to humidity (Sayeed and Benzer, 1996). Hygrotaxis, the ability to migrate up or down a humidity gradient, offers a first line of protection from drying as an animal can simply avoid conditions of desiccation. *C. elegans* exhibits hygrotaxis behavior, a further indication of effective monitoring of humidity (Russell et al., 2014).

Several genes implicated in desiccation resistance suggest an initial outline of some mechanisms involved in hygrosensation. A mutation in a sensory neuron transient receptor potential (TRP) channel gene, *osm-9*, limits desiccation survival (Colbert et al., 1997; Erkut et al., 2013). TRP channels in *Drosophila* antennae regulate hygrosensing, although individual knockdown of *osm-9* or any of the other TRP channels in *C. elegans* does not affect hygrotaxis (Sayeed and Benzer, 1996; Liu et al., 2007; Russell et al., 2014; Enjin et al., 2016). In *C. elegans*, mechanosensory neurons may detect humidity-dependent stretch in the cuticle or epidermis to regulate hygrotaxis (Russell et al., 2014). Additionally, a cGMP gated channel (*tax-4*) in thermosensory neurons regulates hygrotaxis (Russell et al., 2014). It is somewhat surprising that genes like *osm-9* could impact desiccation survival but not hygrotaxis. It is possible that only partially overlapping systems are used for the neuronal regulation of hygrotaxis and the sensing response necessary for desiccation resistance.

Beyond the initial sensing of changes in humidity, this information must be conveyed in some form throughout the organism – likely resulting in altered transcription factor activity and transcriptional regulation of desiccation-related genes. Our understanding of such signaling is sparse, but some clues involve possible roles for a notch ligand (OSM-11), a patched related protein (DAF-6), and mitogen-activated protein kinase (MAPK) signaling (Banton and Tunnacliffe, 2012; Erkut et al., 2013). Specific transcription factors are known to regulate expression of many of the genes and pathways that affect desiccation tolerance. For example, *hsf-1* regulates the heat shock response and many chaperones, *skn-1*/Nrf2 is a master regulator of the oxidative stress response, and *daf-16*/FOXO transcriptionally regulates many genes involved in the glyoxylate shunt and trehalose synthesis during starvation (Hsu et al., 2003; Park et al., 2009; Brunquell et al., 2016; Hibshman et al., 2017). However, there have not yet been any studies specifically testing for a role for some of these well-known transcription factors during desiccation. The tools available in *C. elegans* make it an ideal animal model system to investigate the spatial and temporal nature of signaling required to mount a desiccation response.

### Lessons From Nematodes

The study of desiccation tolerance in a genetic system has facilitated the discovery of more genes associated with desiccation survival than in *Artemia* and tardigrades. More genes than discussed above play roles in desiccation tolerance. These genes (from Erkut et al., 2013) are included in Table 1. Although *C. elegans* studies have unveiled a large number of new molecular players, the mechanisms by which many of these genes contribute to survival remain murky. Further research on *C. elegans* desiccation resistance should be productive toward continued discovery of protectants and delineation of signaling pathways that contribute to survival, and to better understanding mechanisms used by animals more generally. The molecular mechanisms identified in *C. elegans* are likely to be conserved among other nematodes, because studies of gene expression find upregulation during desiccation of similar protectants as described above. For example *Panagrolaimus superbus* increases expression of a small heat shock protein, an LEA protein, and several antioxidants in response to desiccation (Tyson et al., 2012).

## Tardigrades

Tardigrades, also known as water bears, are microscopic eight-legged invertebrates with the ability to survive a wide range of stresses including radiation, temperature stress, high or low pressure, exposure to space, and desiccation (Seki and Toyoshima, 1998; Goldstein and Blaxter, 2002; Jönsson et al., 2008; Møbjerg et al., 2011; Guidetti et al., 2012; Tsujimoto et al., 2016). Although tardigrades are often championed for tolerance of a broad range of extremes, survival of some of these stresses can rely on prior exposure to desiccation. For example, tardigrades like *Ramazzottius varieornatus* are only able to survive at high temperatures when they are in the desiccated state (Neves et al., 2020). Thus, the mechanisms of cellular protection that tardigrades engage during desiccation may explain at least in part the tolerance of other extremes.

In order to survive desiccation, most tardigrades contract into a quiescent tun state. Tun formation involves significant structural changes, including a reduction of surface area that may slow the rate of water loss (Wright, 1989; Halberg et al., 2013; Czerneková et al., 2017; Richaud et al., 2020). Nonetheless, tardigrades lose nearly all intracellular water – meeting the criterion for classification as a true anhydrobiote (Crowe, 1972). In anhydrobiosis, oxygen consumption is reduced to near zero, suggesting complete or nearly complete arrest of metabolism (Pigon and Weglarska, 1955). It is worth noting that while the tun form is most commonly associated with desiccation resistance, there are reports of embryos surviving desiccation, as well as some tardigrades in which tun formation is apparently not required for desiccation survival (Guidetti and Jönsson, 2002; Bertolani et al., 2004; Rebecchi et al., 2006; Schill and Fritz, 2008; Hygum et al., 2016).

Recently, there has been an effort to develop tardigrades as a model for the study of the molecular biology of extremes like anhydrobiosis (see Hashimoto and Kunieda, 2017; Goldstein, 2018 for review). As tardigrades have gained in popularity as a research organism, many studies have focused on transcriptional responses to desiccation in various species (Schill et al., 2004; Reuner et al., 2010; Grohme et al., 2011; Wang et al., 2014; Hashimoto et al., 2016; Boothby et al., 2017; Yoshida et al., 2017). In contrast, few studies test for endogenous functions of proteins in tardigrades (Tenlen et al., 2013; Boothby et al., 2017). RNAi for gene knockdown has been demonstrated in a common lab-reared species, *Hypsibius exemplaris* (recently disambiguated from the closely related species *Hypsibius dujardini*) (Tenlen et al., 2013; Gasiorek et al., 2018; McNuff, 2018). However, development of further genetic methods and implementation in other species of tardigrades will be instrumental for progress in understanding the endogenous roles of tardigrade proteins.

### Varying Dependence on Trehalose

Despite most known anhydrobiotes producing trehalose as a protective mechanism, not all tardigrades are reliant on trehalose (Crowe, 2002). Some tardigrades like *Adorybiotus coronifer* and those of the family Macrobiotidae have been shown to produce trehalose in response to desiccation (Westh and Ramløv, 1991; Hengherr et al., 2008; Jonsson and Persson, 2010). Other species like *E. granulatus* and *E. testudo* synthesize low quantities of trehalose but do not upregulate synthesis during desiccation (Hengherr et al., 2008; Jonsson and Persson, 2010). Other tardigrades, like *H. exemplaris*, appear to lack entirely the trehalose 6-phosphate synthase and trehalose 6-P phosphatase enzymes required for trehalose synthesis (Cesari et al., 2012; Boothby et al., 2017; Yoshida et al., 2017). Rotifers are also able to survive desiccation without synthesis of trehalose, suggesting that tardigrades are not unique in this respect (Lapinski and Tunnacliffe, 2003; Tunnacliffe et al., 2005). Even in tardigrades that produce trehalose, the maximum amount reported (2.9% of the dry weight in anhydrobiotic *Macrobiotus islandicus*) is considerably lower than the level of trehalose accumulation reported in *Artemia* (15%) or nematodes (∼13%) (Clegg, 1965; Madin and Crowe, 1975; Crowe et al., 1977; Jonsson and Persson, 2010). The apparent lack of a requirement for trehalose in desiccation tolerance of some tardigrades indicates that other molecules must have protective effects that substitute for the function of trehalose.

### Novel Disordered Proteins: MAHS, CAHS, SAHS

Protectants that function during desiccation must retain their protective properties even during conditions in which most proteins are prone to aggregation and dysfunction. Thus, protective proteins are likely to be resistant to aggregation and retain solubility even during extreme conditions if they are to interact with and preserve other cellular constituents. If protectants that function during desiccation are themselves resistant to severe dehydration stress, this may predispose them to withstand other stresses as well. To identify possible protectant proteins, one approach has been to determine the portion of the proteome that is highly stable and soluble even during stress. The most straightforward method for this has been to search for heat soluble proteins – those that can retain solubility even after transient exposure to near-boiling temperatures. Analysis of the tardigrade proteome has revealed such heat soluble proteins (Yamaguchi et al., 2012).

Tardigrades possess several classes of heat stable proteins: cytosolic abundant heat soluble (CAHS), mitochondrial abundant heat soluble (MAHS), and secretory abundant heat soluble (SAHS) proteins (Yamaguchi et al., 2012; Tanaka et al., 2015). The SAHS proteins have structural similarity to fatty acid binding proteins, but the CAHS proteins do not contain any conserved domains or strong homology to other proteins (Yamaguchi et al., 2012; Fukuda et al., 2017). These proteins are thus likely to be unique to tardigrades, and as a result, they have also been referred to as tardigrade-specific intrinsically disordered proteins (TDPs) (Boothby et al., 2017). Such proteins were first identified from experiments in *Ramazzotius varieornatus*, and homologs for some of the heat soluble proteins are also present in other tardigrade species including *H. exemplaris* and *Milnesium tardigradum* (Yamaguchi et al., 2012). Further, some TDPs are transcriptionally upregulated in response to desiccation in *H. exemplaris* and *P. richtersi* (Boothby et al., 2017). Some CAHS proteins show protective effects outside of their normal context in tardigrades: they are sufficient to increase desiccation tolerance of bacteria and yeast, and they can protect an enzyme (lactate dehydrogenase) in solution from desiccation-induced inactivation (Boothby et al., 2017). RNAi knockdown of some CAHS or SAHS proteins reduces desiccation survival in tardigrades (Boothby et al., 2017). These experiments identify extremotolerance protectants that are not only sufficient in heterologous cells or in solution, but that also can be demonstrated to function *in vivo*.

The potentially broad function of TDPs piques interest in understanding the mechanisms by which they exert their protective effects. As with LEA proteins, the disordered nature of CAHS proteins and other TDPs goes against traditional intuition of structure-function analysis. However, CAHS proteins take on a glass form during desiccation, suggesting that disordered proteins in general may contribute to cellular vitrification – a mechanism we will discuss in more detail below (Boothby and Pielak, 2017; Boothby et al., 2017; Janis et al., 2018).

### LEA Proteins

As well as novel TDPs, tardigrades also have genes encoding group 3 LEA proteins (Förster et al., 2009, 2012; Schokraie et al., 2010; Tanaka et al., 2015). Heterologous expression in cultured human cells, a similar approach to that used by *Artemia* researchers, was used along with immunolocalization in tardigrades to determine the subcellular localization of a group 3 LEA protein of *Ramazzottius varieornatus*, RvLEAM. This LEA is mitochondrially localized, similar to AfrLEA3m (Tanaka et al., 2015). Thus, distribution of LEA proteins to different subcellular regions may represent a widespread strategy that contributes to cellular survival.

### Heat Shock Proteins

Heat shock proteins in tardigrades have been identified in several studies, and in some cases have been shown to be upregulated during anhydrobiosis (Reuner et al., 2010; Schokraie et al., 2010, 2011; Förster et al., 2012). The full repertoire of heat shock proteins seems to be conserved in tardigrades, including sHSPs and Hsp70 (Förster et al., 2009, 2012; Yoshida et al., 2017). Some of the small heat shock proteins (like Hsp27 and Hsp30c of *M. tardigradum*) are also upregulated during dehydration (Wang et al., 2014). In contrast, Hsp70 expression is not increased during desiccation, but is instead upregulated during rehydration and recovery (Schill et al., 2004; Jönsson and Schill, 2007; Rizzo et al., 2010; Altiero et al., 2012). This expression timeline is consistent with a possible role for Hsp70 in folding or refolding proteins to restore proteostasis following desiccation.

### Oxidative Stress Response

Oxidative stress has been proposed as a factor that ultimately limits the time tardigrades can survive desiccation (Guidetti and Jönsson, 2002). There is evidence for increased superoxide dismutase activity and peroxidase activity during desiccation, as well as an overall increase in levels of the reducing agent glutathione (Rizzo et al., 2010). These changes likely counter the accumulation of oxidative damage during desiccation.

### DNA Preservation

Extended time spent in anhydrobiosis leads to DNA damage, suggesting a genotoxic effect of desiccation (Neumann et al., 2009). However, short-term desiccation does not cause significant DNA damage, suggesting that animals are able to sufficiently protect DNA during the early stages of desiccation (Rebecchi et al., 2009). Tardigrades are also able to survive exposure to relatively high doses of UV, X-ray, and gamma radiation (Horikawa et al., 2013; Beltrán-Pardo et al., 2015; Hashimoto and Kunieda, 2017). These observations suggest that tardigrades exhibit DNA protection mechanisms that may function during anhydrobiosis. To preserve the integrity of their genome, tardigrades must either prevent or efficiently repair DNA damage. Components of traditional DNA damage repair pathways have been found to be present in many tardigrade species, and a novel tardigrade protein, Dsup, has been identified that suppresses DNA damage (Hashimoto et al., 2016; Yoshida et al., 2017; Carrero et al., 2019; Kamilari et al., 2019). Dsup binds to DNA and prevents fragmentation in response to X-ray irradiation, H_2_O_2_, and hydroxyl radicals (Hashimoto et al., 2016; Hashimoto and Kunieda, 2017; Chavez et al., 2019). Expression of Dsup in cultured human cells can improve survival of X-ray irradiation and enable subsequent proliferation (Hashimoto et al., 2016). Dsup has been investigated primarily for a role in preventing DNA damage caused by specific genotoxic agents, but it could feasibly function similarly during desiccation to preserve the integrity of DNA.

### Lessons From Tardigrades

As with research on *Artemia*, heterologous systems and *in vitro* studies have predominantly fueled our understanding of the functions of tardigrade proteins. Studies of endogenous functions of tardigrade proteins have been rare. This is likely due to the limited resources currently available for reverse genetics. To date, use of RNAi for gene knockdown has been documented in only one species of tardigrade, *H. exemplaris* (Tenlen et al., 2013). Expanding such methods to other species of tardigrades will allow for more studies of endogenous functions. The lack of reliance on trehalose for desiccation tolerance and discovery of novel TDPs suggest that further mechanisms likely remain to be uncovered.

## Common Mechanisms of Desiccation Tolerance

Several common themes of desiccation tolerance have emerged from research in *Artemia*, nematodes, and tardigrades. For example, each of these three animals has genes encoding LEA proteins and heat shock proteins like Hsp70 and small heat shock proteins (see Table 1). Other mechanisms are more prominent in some animals than others. For example *Artemia* and *C. elegans* produce significant levels of trehalose, while some species of tardigrades produce none at all. In many cases, proteins found to function in one of these systems simply have not been tested in others. For example, Hsf1 is required for desiccation resistance in *Artemia* but, to date, has not been explored in either tardigrades or nematodes. In such cases research from other organisms can supplement our understanding. For example Hsf1 of the anhydrobiotic midge *Polypedilum vanderplanki* is responsible for regulation of desiccation-responsive transcripts (Mazin et al., 2018). Finding and filling these knowledge gaps across organisms will inform the evolutionary history of desiccation resistance and reveal the extent to which regulation of the desiccation response and the protectants that allow for survival are conserved amongst anhydrobiotes. Looking beyond individual protectants, there are several generalizable mechanisms (often involving some of the molecular players already discussed) that have been proposed. Figure 2 depicts some of the common protectants and mechanisms that have been identified and their organization within a cell. So far, three key biochemical models for desiccation tolerance have emerged.

### Water Replacement Hypothesis

In hydrated cells, water provides a polar solvent in which molecules with exposed hydrophilic regions are stable. The loss of water during desiccation disrupts the normal chemical interactions that maintain proteostasis, membrane stability, and other essential cellular functions. The hypothesis was put forward that by synthesizing large quantities of polar molecules like glycerol or trehalose, perhaps cells are able to simulate the types of molecular interactions (like hydrogen bonding) that would occur in an aqueous environment (Crowe and Clegg, 1973; Clegg et al., 1982). A particular example of this is the preservation of lipid bilayers by trehalose during desiccation (see Figure 2) (Crowe et al., 1984). In hydrated conditions, water surrounds phospholipid head groups, providing spacing between these polar (hydrophilic) groups (Crowe et al., 1998b). Loss of water molecules as an insulator between these phospholipids causes compression of the membrane leading to increased rigidity and ultimately fragmentation and leakage (Crowe et al., 1984, 1998b). Trehalose buffers membranes from these effects by substituting for water and associating with phospholipid head groups, thereby providing adequate spacing of phospholipids and allowing for retention of membrane fluidity (Crowe et al., 1998b; Golovina et al., 2010; Abusharkh et al., 2014). Trehalose can also preserve the morphology of lipid raft domains in drying membranes (Chiantia et al., 2005). The same types of beneficial interactions that preserve membranes may similarly protect proteins that display polar surfaces (Jain and Roy, 2009). It has also been suggested that sugars like trehalose could assist in trapping any residual water and creating a film at critical hydrophilic contact sites (Belton and Gil, 1994; Cottone et al., 2002). The function of trehalose as a “water replacement” likely overlaps with its function in forming biological glasses (Koster et al., 1994; Crowe et al., 2001, 2011; Crowe, 2007).

### Vitrification

A glass is an amorphous, non-crystalline solid. Through the process of vitrification, biological glasses form and protect cells from damage during dehydration (Sun and Leopold, 1997; Crowe et al., 1998a). During drying, vitrification creates a highly viscous matrix that spatially restricts cellular components such that chemical reactions are significantly limited (Buitink et al., 1998). This has been suggested as a means for promoting desiccation-associated metabolic quiescence (Bruni and Leopold, 1991). The process of vitrification is temperature-dependent, and different molecules have unique glass transition temperatures (Crowe et al., 1998a). Trehalose has garnered particular attention for its glass-forming properties (Crowe et al., 2001; Crowe, 2007). While trehalose is not unique in its glass-forming ability, it has a higher glass transition temperature than other sugars – meaning that only at high temperatures will it “melt” and dissolve the glass into a more fluid state (Green and Angell, 1989; Crowe et al., 1998a). In addition to sugars, some proteins have been shown to contribute to glass formation. LEA proteins contribute to biological glass formation and can act synergistically with trehalose to confer desiccation tolerance (Wolkers et al., 2001; Shimizu et al., 2010). Disordered CAHS proteins from tardigrades can also vitrify (Boothby et al., 2017).

Evidence for glassy solids in plants and animals, including *Artemia* and tardigrades, suggest that glass formation is a widespread mechanism contributing to desiccation tolerance (Williams and Leopold, 1989; Sakurai et al., 2008; Hengherr et al., 2009, 2011a; Boothby et al., 2017). While some examples of the molecules that allow for vitrification have been discovered, there are likely more components that contribute to cellular glass formation that remain to be uncovered. Our understanding of the physiology of vitrification will grow as organismal and biochemical studies converge.

### Molecular Shielding

The term “molecular shield” was first proposed to describe the function of LEA proteins that may provide a protective shell that shields proteins during desiccation and prevent aggregation (Wise and Tunnacliffe, 2004; Goyal et al., 2005). The proposed function of a molecular shield protein is to limit the number and frequency of intermolecular interactions that could lead to protein aggregation during desiccation (Liu et al., 2011; Chakrabortee et al., 2012; Hatanaka et al., 2013). This function is similar to that of classic molecular chaperones like some HSPs – for example small heat shock proteins that can non-enzymatically limit protein aggregation by creating a similar “shield” around misfolded proteins and prevent interactions with neighboring proteins that would otherwise coalesce into aggregates (Tunnacliffe and Wise, 2007; Haslbeck et al., 2019). Notably, molecular shielding has been reserved primarily as a descriptor for the function of disordered proteins with non-specific binding partners (Chakrabortee et al., 2012). Whereas some chaperones like Hsp70 specifically bind to client proteins that need to be refolded, LEAs and other shield proteins are thought to bind less specifically, but rather offer a general buffer by slowing the rate of protein-protein interaction that may lead to aggregation (Liu et al., 2011; Chakrabortee et al., 2012).

### Mechanistic Synergy

While the water replacement hypothesis, vitrification, and molecular shielding are sometimes discussed as individual mechanisms for desiccation survival, they likely overlap and synergistically promote desiccation tolerance. This can be appreciated when individual molecular protectants contribute to more than one of these models. For example, LEA proteins contribute to each of the three models for protection, and trehalose plays a prominent role in both the water replacement hypothesis and vitrification (Hand et al., 2011). Thus, fitting individual protectants into a single mechanistic model may be too simplistic. Further, some studies have found synergy between protectants like trehalose, IDPs, p26, and LEA proteins (Goyal et al., 2005; Ma et al., 2005; Kim et al., 2018). The three models discussed share some common protectant molecules and may in fact be largely one coherent mechanism. For example, as LEA proteins are incorporated into amorphous biological glasses, they are dispersed throughout the cell and can slow molecular interactions as they shield aggregation-prone proteins in a glassy matrix, and at the same time provide stability to polar molecules with their amphipathic or hydrophilic structure (Tunnacliffe and Wise, 2007).

## Discussion

### Conclusion

Our knowledge of the mechanisms of desiccation tolerance has been largely informed by research on a small number of anhydrobiotic organisms. Work on *Artemia*, nematodes, and tardigrades has advanced our understanding of the protectants and mechanisms that contribute to desiccation survival. Comparisons of the types of protectants involved in desiccation tolerance in each animal converges on a few key molecules (see Table 1). However, there are noteworthy variations to these common molecular and mechanistic themes: examples like tardigrades apparently lacking trehalose and the *Artemia*-specific protein, Artemin. Each organism has brought with it unique experimental advantages as well – *Artemia* have a unique life cycle and intergenerational diapause, *C. elegans* has provided a genetic model to greatly facilitate gene identification and functional studies, and tardigrades are emerging as another model for exceptional stress tolerance. A comparative organismal approach to the study of desiccation tolerance, using these organisms and others such as rotifers and midges, has enriched our understanding of mechanisms that multicellular organisms employ to survive desiccation, and no doubt will continue to do so.

### Open Questions

Desiccation tolerance is sometimes conceived as primarily a cellular survival strategy. While individual cells must synthesize the necessary protectants to survive, the coordination of the desiccation response in multicellular organisms presents an expanded set of challenges – to sense and respond appropriately to environmental cues that signal dehydration, and coordinate a response across tissues to allow the animal to survive. This necessitates exquisite spatial and temporal regulation. While some mechanisms by which this sensing and coordination are accomplished have been discussed, the basis of the intermediate regulatory steps between survival of an organism and survival of a cell remain largely unknown.

In many cases, heterologous expression of proteins demonstrates the possible activity of protectants for desiccation survival. However, the endogenous functions of proteins are often poorly characterized. Conducting more *in vivo* experiments and drawing conclusions about protectants in their native context will help to distinguish between possible protectants and those that are essential. Still, part of the promise in studying mechanisms of desiccation tolerance is the application of discoveries to better preserve biomaterials. Therefore, discovery of new, uncharacterized protectants continues to be of interest. In over 300 years since the initial observation of desiccation survival, our understanding has grown considerably. Yet, in many ways the molecular basis of anhydrobiosis and mechanisms by which multicellular animals survive drying are only beginning to emerge. Continued research in *Artemia*, nematodes, and tardigrades in coming years will likely contribute significantly to our growing understanding of desiccation tolerance.

## Author Contributions

JH drafted the original manuscript. All authors edited and revised the manuscript, approved the final version, and contributed to the conception of this review and discussion of its contents.

## Conflict of Interest

The authors declare that the research was conducted in the absence of any commercial or financial relationships that could be construed as a potential conflict of interest.
